# Hsa-miR-181a-5p Enhances the Chemosensitivity of Breast Cancer Cells to Tamoxifen by Regulation of the OSBPL3/RAS Signaling Pathway

**DOI:** 10.7759/cureus.94996

**Published:** 2025-10-20

**Authors:** Jianmin Lin, Xiujuan Li, Xiaojiao Qu, Chenxi Li, Sijia Cao, Xiaochun Fu

**Affiliations:** 1 Department of Laboratory Medicine, Fujian Key Clinical Specialty of Laboratory Medicine, Women and Children’s Hospital, School of Medicine, Xiamen University, Xiamen, CHN; 2 Department of Cardiac Surgery, Cardiovascular Hospital, School of Medicine, Xiamen University, Xiamen, CHN

**Keywords:** breast cancer research, hsa-mir-181a-5p, osbpl3, signaling pathway, tamoxifen

## Abstract

Background: Despite tamoxifen being a broad-spectrum therapeutic agent for breast cancer (BC) management, the emergence of chemoresistance significantly compromises its clinical effectiveness. OSBPL3 has been identified as a metastasis-promoting protein implicated in BC progression, yet the upstream regulatory mechanisms controlling its expression remain poorly understood, particularly regarding microRNA-mediated regulation.

Methods: MCF-7 cells underwent lentiviral infection for stable gene modification, followed by transient transfection with hsa-miR-181a-5p mimics (or corresponding negative controls), antisense miR-181a-5p inhibitors (anti-miR-181a-5p), and OSBPL3-specific small interfering RNAs (siRNAs). The direct binding interaction between miR-181a-5p and OSBPL3 was validated using a dual-luciferase reporter system containing wild-type or mutant 3'UTR sequences. Tamoxifen sensitivity was evaluated through functional assessments, including CCK-8 assay, Annexin V-FITC/PI apoptosis assay, and Transwell migration/invasion assays. Gene expression was assessed by Western blot analysis.

Results: Our findings revealed that miR-181a-5p inhibits OSBPL3 expression by specifically targeting the 3’-UTR of OSBPL3. At the pharmacologically active concentration of 5 µM tamoxifen, overexpression of miR-181a-5p led to the suppression of OSBPL3 expression, which in turn inhibited the invasion, migration, and proliferation of BC cells, while increasing their apoptosis. These effects were further enhanced when OSBPL3 was concurrently interfered with. Additionally, knockdown of OSBPL3 in MCF-7 cells resulted in the coordinated downregulation of five positive regulators of RAS signaling, leading to the activation of the RAS signaling pathway.

Conclusions: These results suggested that the upregulation of miR-181a-5p enhanced chemosensitivity to tamoxifen by negatively regulating OSBPL3 through the RAS signaling pathway in BC cells. Therefore, a treatment strategy based on the miR-181a-5p/OSBPL3 axis may represent a potential approach to overcoming tamoxifen resistance in BC.

## Introduction

Breast cancer (BC) is the most prevalent cancer among women globally, accounting for approximately 30% of all new female cancer cases [1]. In China, BC mortality ranks first among women with malignant tumors, and it is the second leading cause of cancer-related death for women worldwide, accounting for roughly 15% of all female cancer deaths [2,3]. Nearly 70% of BC patients are estrogen receptor (ER)-positive (ER+) [4], making them eligible for endocrine therapies such as tamoxifen, fulvestrant, and letrozole [5]. Tamoxifen, a selective estrogen receptor modulator, competitively binds to the ER and inhibits ER-induced BC cell growth. As an effective antiestrogenic drug, tamoxifen is widely used as the standard therapy for ER+ BC patients. However, the acquisition of tamoxifen resistance poses a significant challenge during endocrine therapy [6]. Several biological mechanisms of tamoxifen resistance have been reported, including mutations/deletions in estrogen receptor 1 (ESR1) and the activation of other cell proliferation pathways [7-9]. Nevertheless, there is an urgent need to identify additional molecular mechanisms underlying tamoxifen resistance.

As an emerging regulator within the oxysterol-binding protein (OSBP) family, OSBPL3 has garnered increasing attention for its multifaceted biological roles [10]. This dual-function protein serves not only as an intracellular lipid receptor/transporter governing cholesterol homeostasis [11], but also as a signaling scaffold that coordinates vesicular trafficking, cytoskeletal remodeling, and mitogenic pathway activation [12]. Mounting evidence implicates OSBPL3 dysregulation in oncogenic processes, where its aberrant expression drives cell cycle acceleration and enhances metastatic competence through epithelial-mesenchymal transition (EMT) activation [13,14]. However, the underlying mechanism by which the upstream regulators of OSBPL3 modulate its expression remains to be elucidated, with particularly limited insight into post-transcriptional control mechanisms mediated by tumor-suppressive microRNAs (miRNAs).

MicroRNAs, a class of non-coding RNAs comprising 17-25 nucleotides, facilitate post-transcriptional regulation through sequence-specific Watson-Crick base pairing with complementary sequences in the 3' untranslated regions (3'UTRs) of target mRNAs, thereby leading to translational repression or transcript destabilization [15]. A growing body of evidence suggests that dysregulated miRNA expression plays pivotal roles in various human cancers, frequently acting as critical tumor suppressors or oncogenes to influence breast tumorigenesis and progression [16-19]. In this study, the interaction between miR-181a-5p and the 3'-UTR of OSBPL3 was validated using bioinformatics predictions and dual-luciferase reporter assays. Quantitative reverse transcription polymerase chain reaction (qRT-PCR) and Western blot analyses demonstrated that miR-181a-5p regulates OSBPL3 expression. In vitro experiments revealed that overexpression of miR-181a-5p enhances the chemosensitivity of BC cells to tamoxifen by inhibiting OSBPL3. Furthermore, miR-181a-5p was observed to concurrently downregulate five positive regulators of the RAS signaling pathway, leading to its activation. Collectively, these findings suggest that the miR-181a-5p/OSBPL3 axis may provide new predictive biomarker pathways and represent a novel molecular target for the prevention and treatment of BC metastasis.

## Materials and methods

Oligonucleotides, plasmid, and transfection

The miR-181a-5p mimics/inhibitors, along with small interfering RNAs (siRNAs) targeting OSBPL3 and their respective controls, were purchased from RiboBio (Guangzhou, China). The oligonucleotide sequences are detailed in the Appendices. Sequences containing predicted miRNA binding sites or their corresponding mutants were synthesized and subsequently cloned into the psiCHEK2 vector (Promega, Madison, WI) for use in miRNA target gene luciferase reporter assays.

Cell culture

The human breast cancer MCF-7 (HTB-22) and HEK-293T (CRL-3216) cell lines were sourced from the American Type Culture Collection (ATCC). These cell lines were cultured in Dulbecco’s Modified Eagle Medium (DMEM) (HyClone, Logan, UT) supplemented with 10% fetal bovine serum (Gibco, Waltham, MA) and 100 units/ml penicillin/streptomycin (Gibco). The cells were maintained at 37°C in a humidified atmosphere with 5% CO2.

Formulation of the Lenti-DNA-Mix transfection system

The Lenti-DNA-Mix transfection system was formulated by adding the control plasmid, the interfering plasmid, and the lentiviral packaging plasmid to HEK-293T cells. The total amount of plasmid DNA added was 24 μg, consisting of 12 μg of lentiviral plasmid and 12 μg of Lenti-Mix (with a ratio of pMDLg/pRRE:pVSV-G:pRSV-Rev = 5:3:2). The plasmids were added dropwise to the cells, which were then placed in a 37°C cell culture incubator containing 5% CO2. After 48 hours of transfection, the cell culture medium was collected and centrifuged at 3000×g for 10 minutes at 4°C. The supernatant was subsequently filtered through a 0.45 μm filter to obtain the lentiviral supernatant. This supernatant could be stored at 4°C for short-term use or frozen at -80°C for long-term storage, avoiding repeated freezing and thawing.

Lentivirus infection

MCF7 cells were collected by trypsinization and plated in complete medium at a density of 5×105 cells/cm² on a six-well cell culture dish. A total of 100 μL of lentiviral supernatant and 1900 μL of complete medium were added, followed by polybrene to achieve a final concentration of 10 μg/mL. After 24 hours, the lentivirus-containing medium was removed and replaced with fresh complete medium, and the cells were allowed to continue being infected for 72 hours. Subsequently, 2 μg/mL puromycin was added for 48 hours to establish gene knockdown BC cells.

Cell transfection

MCF7 cells were transfected with miR181a mimics, mimics-NC (negative control), miR-181a inhibitor, inhibitor NC, and OSBPL3-lentivector using Lipofectamine® 3000 (Thermo Fisher Scientific, Waltham, MA) according to the manufacturer's instructions. The lentivirus used was sourced from Shanghai, China, and the cells were divided into seven experimental groups: miRNA mimics NC, miRNA mimics, miRNA inhibitor NC, miRNA inhibitor, miRNA mimics NC and interference NC, OSBPL3 interference, and miRNA mimics and OSBPL3 interference. All sequences utilized in the study are provided in the Appendices.

Measurement of cell growth

Cell proliferation was measured by using a Cell Counting Kit-8 (MCE, China). MCF7 cells were seeded at 0.6 × 104 cells per well in a 96-well plate and cultured overnight. The cells were then treated with various concentrations (0, 5, 10, 20, and 40 µM) of tamoxifen (MedChemExpress, Monmouth Junction, NJ; dissolved in DMSO) and were incubated for 48 hours. Cell proliferation was determined by CCK-8 solution, and the optical density was measured at 450 nm.

qRT-PCR

Total mRNA was extracted using the TRIzol Reagent Kit, and reverse transcription was performed using the PrimeScript RT Reagent Kit (Takara Bio, Inc., Shiga, Japan). miRNA was isolated using the miRNA Extraction Kit (Tiangen Bio, Shanghai, China), and the expression levels of mature miRNAs were determined using stem-loop reverse transcription. Gene expression was quantified using a qRT-PCR system (StepOne Plus, Applied Biosystems, Waltham, MA). The relative expression of miRNA or target genes was normalized to U6 or GAPDH expression, respectively. The 2−ΔΔCt method was used to calculate the relative expression levels. The primers utilized in this study are listed in the Appendices.

Western blot analysis

BC cells were harvested during the logarithmic growth phase, and total protein was extracted using radioimmunoprecipitation assay (RIPA) buffer (Sangon Biotech Co., Ltd., Shanghai, China). Protein concentration was subsequently determined using a bicinchoninic acid (BCA) kit (Sangon Biotech Co., Ltd.). Following 12% SDS-PAGE (sodium dodecyl sulfate-polyacrylamide gel electrophoresis), protein samples (30 µg per well) were transferred onto polyvinylidene difluoride (PVDF) membranes and blocked with 5% bovine serum albumin (BSA) (Sigma-Aldrich, Merck KGaA, St. Louis, MO) at room temperature for one hour. The membranes were then incubated overnight at 4°C with primary antibodies against GAPDH (dilution 1:3,000, AC002, Abclonal), OSBPL3 (dilution 1:1,000, A4604, Abclonal), ERK1/ERK2 (dilution 1:1,000, A16686, Abclonal), p-ERK1/ERK2 (dilution 1:1,000, AP0472, Abclonal), AKT (dilution 1:1,000, A18675, Abclonal), p-AKT (dilution 1:1,000, AP0637, Abclonal), and Ras (dilution 1:1,000, ab52939, Abcam). After washing the membranes three times with Tris-buffered saline with Tween (TBST) (10 minutes per wash), they were incubated with the corresponding horseradish peroxidase (HRP)-labeled secondary antibodies (dilution 1:5,000, ab6721, Abcam) for one hour at room temperature. Immunoblots were visualized using an electrochemiluminescence (ECL) system (EMD Millipore, Burlington, MA), and densitometry was performed using a multimode microplate reader (Varioskan LUX, Thermo Fisher Scientific).

Wound-healing assay

BC cells were seeded into a six-well plate, and when the cell confluence reached 60%-80% in the different groups, a scratch was made in the monolayer using a pipette tip. Floating cells were then removed using phosphate-buffered saline (PBS). The remaining adherent cells were cultured in serum-free medium to support cell growth and facilitate wound healing. Images were captured using a phase contrast microscope (Olympus, Tokyo, Japan) at 0 and 48 hours after culturing.

Transwell assay

The Matrigel (diluted 1:5; BD Biosciences, Franklin Lakes, NJ) was prepared using a serum-free medium, and 100 μL of this diluted Matrigel was incubated at 37°C for five hours. Following the manufacturer's protocols, the transwell chamber was positioned on a 24-well plate, with the lower chamber containing 500 μL of medium supplemented with 10% fetal bovine serum (FBS). The treated cells were washed with PBS, resuspended in serum-free medium, and 200 μL of serum-free medium containing 2×105 cells was added to each chamber. After 48-hour incubation, BC cells that had migrated to the lower layer were fixed, stained with crystal violet, and visualized using microscopy.

Apoptosis assay

For the apoptosis assay, BC cells were collected and stained with propidium iodide (PI) and Annexin V-FITC, following the manufacturer's instructions (Sangon Biotech Co., Ltd.). The stained cells were subsequently analyzed via flow cytometry (Guava EasyCyte; InCyte Software; EMD Millipore). All experiments were conducted in triplicate.

Luciferase reporter assay

To predict the regulatory role of miR-181a-5p on OSBPL3, a target reporter plasmid containing either the wild-type (WT) or mutant 3’-untranslated region (3’-UTR) of OSBPL3 was employed in a luciferase reporter assay. 293T cells were cultured in 24-well plates and co-transfected with 100 ng of either the wild-type or mutated OSBPL3 3’-UTR constructs, along with negative control (NC) or miR-181a-5p mimics, utilizing Lipofectamine 3000 (Invitrogen, Thermo Fisher Scientific) according to the manufacturer's instructions. Luciferase activity was assessed 48 hours post-transfection using a dual luciferase reporter assay kit (E1910, Promega, Madison, WI). Firefly luciferase activity was normalized to Renilla luciferase activity.

Statistical analysis

All experiments were conducted in triplicate. The data are presented as mean ± standard deviation (SD) and were analyzed using SPSS version 19.0 (IBM Corp., Armonk, NY). One-way analysis of variance (ANOVA) followed by Tukey's honestly significant difference (HSD) test was performed to determine significant differences between or among groups. A p-value less than 0.05 was deemed statistically significant.

## Results

Short hairpin RNA (shRNA) OSBPL3-2 was selected for cellular experiments

**Figure 1:**
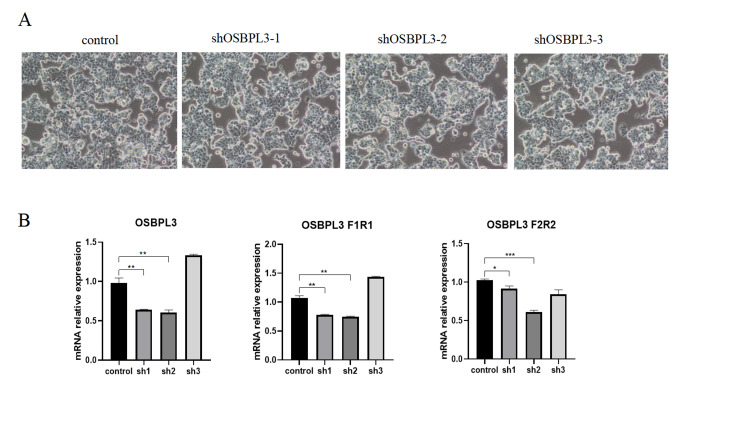
Construction of OSBPL3 knockdown breast cancer cells. (A) Morphology of OSBPL3 knockdown MCF7 cells. (B) The expression level of shOSBPL3 in the three interference groups was significantly down-regulated. Data were reported as mean ± standard deviation of three independent experiments. * P < 0.05, ** P < 0.01, and *** P < 0.001.

To ensure effective interference, given that the transient transfection efficiency of plasmids into MCF7 cells did not exceed 90%, we employed a lentiviral-mediated RNA interference system to generate stable OSBPL3-knockdown BC cells. The established MCF7-derived cell lines included a negative control group infected with a negative lentiviral and three experimental groups infected with distinct OSBPL3-targeting shRNA lentiviral (Figure 1A). qRT-PCR analysis was performed to evaluate the knockdown efficiency. Data demonstrated that the shOSBPL3-2 group exhibited the most significant suppression of OSBPL3 mRNA expression (Figure 1B). Based on this optimal silencing efficacy, the shOSBPL3-2 lentiviral was subsequently utilized for functional investigations.

Has-miR-181a-5p enhanced the tamoxifen chemosensitivity in MCF7 cells

**Figure 2:**
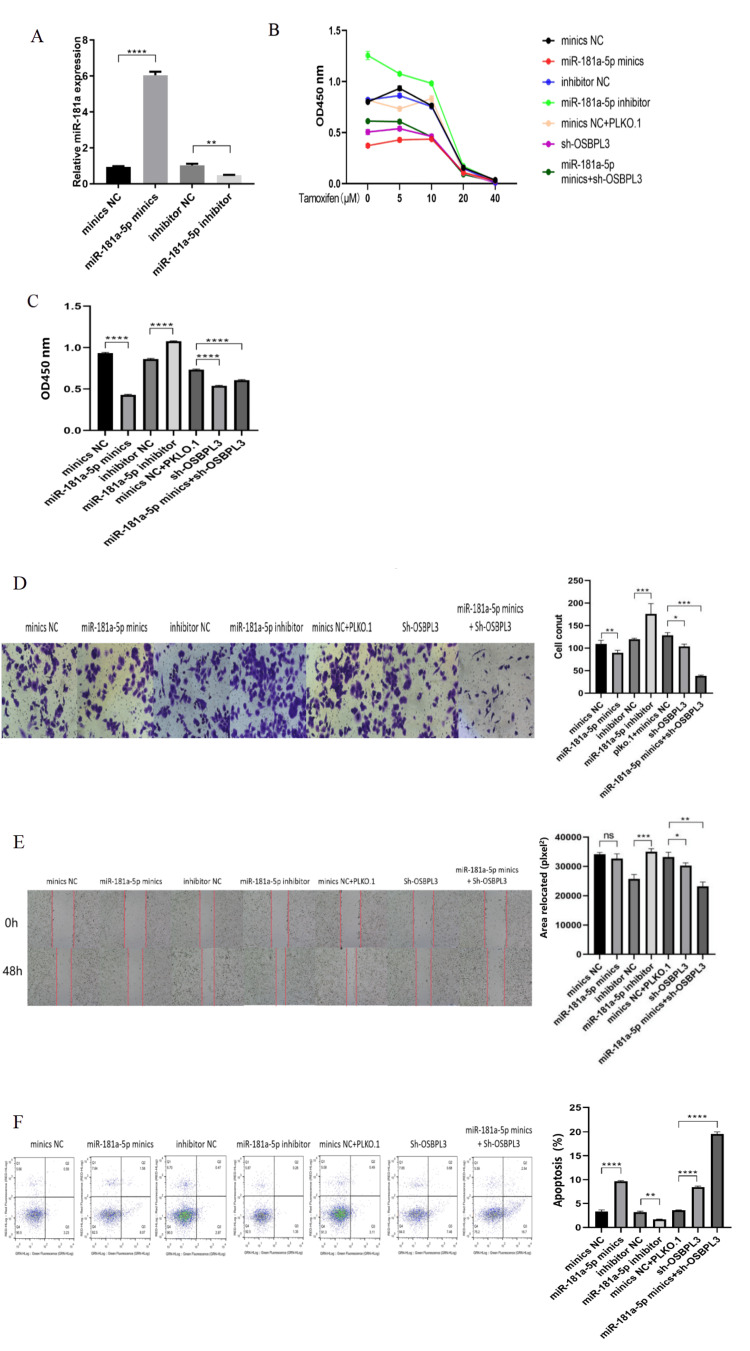
miR-181a-5p enhanced tamoxifen chemosensitivity in MCF7 cells. (A) miR-181a-5p expression among the groups. (B) Cell proliferation was determined using a Cell Counting Kit-8 assay with various concentrations of tamoxifen. (C) Cell proliferation with 5 µM tamoxifen. (D) Cell invasion ability was determined using a Transwell assay. (E) Cell migratory abilities were determined using a wound-healing assay. (F) Cell apoptosis was determined by flow cytometry. Data were reported as mean ± standard deviation of three independent experiments. * P < 0.05, ** P < 0.01, *** P < 0.001, and **** P < 0.0001 vs. control group. NC, negative control.

To elucidate the mechanistic role of miR-181a-5p in modulating tamoxifen therapeutic efficacy through regulation of proliferation, migration, and apoptosis, MCF7 cells were transfected with miR-181a-5p mimics, antisense inhibitors, or scramble controls, followed by pharmacological treatment with tamoxifen at escalating concentrations (0, 5, 10, 20, and 40 µM) for 48 hours. qRT-PCR analysis confirmed successful transfection efficiency, with significant differential miR-181a-5p expression across experimental cohorts prior to tamoxifen exposure (Figure 2A). Post-treatment cell viability assessment via CCK-8 assay revealed concentration-dependent responses, with the 5 µM tamoxifen demonstrating the most pronounced intergroup variability (Figure 2B). This concentration was therefore selected for subsequent functional validation.

Comparative analysis at 5 µM tamoxifen demonstrated marked suppression of cell viability in the miR-181a-5p mimic cohort versus controls, whereas partial restoration of proliferative capacity was observed in the inhibitor-treated group (Figure 2C). Functional phenotyping further revealed that miR-181a-5p inhibition significantly enhanced cellular invasion and migration, whereas mimic transfection attenuated these metastatic properties (Figure 2D). Flow cytometric analysis of apoptosis demonstrated that miR-181a-5p overexpression synergistically enhanced tamoxifen-mediated apoptotic response, while inhibition of miR-181a-5p substantially blunted drug-induced apoptosis (Figure 2F).

OSBPL3 functioned as a direct downstream target of miR-181a-5p in MCF-7 cells

**Figure 3:**
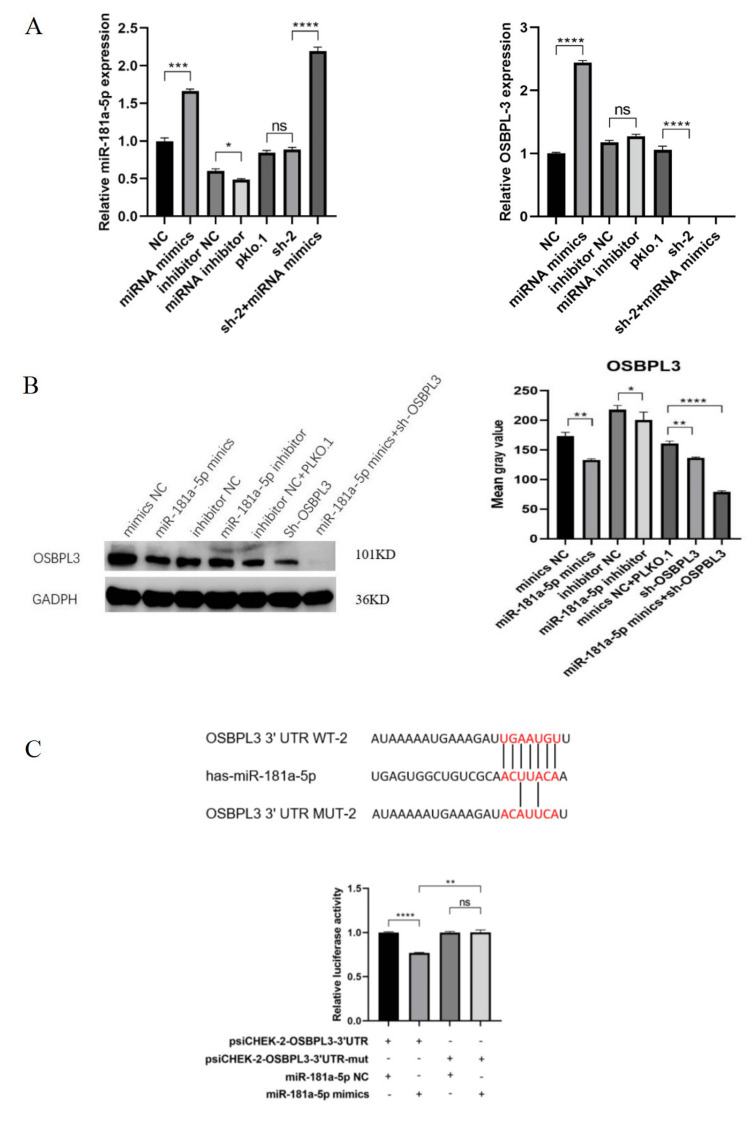
OSBPL3 served as the downstream target of miR-181a-5p in MCF-7 cells. (A) miR-181a-5p and OSBPL3 expression among the groups was determined by quantitative reverse transcription polymerase chain reaction. (B) OSBPL3 expression determined by Western blotting. (C) Luciferase reporter assays in MCF-7 cells after co-transfection with wild-type or mutated 3ʹUTR OSBPL3 plasmids. Luciferase activities were detected 48 hours after transfection. * P < 0.05, ** P < 0.01, *** P < 0.001, and **** P < 0.0001 vs. control group. NC, negative control.

Bioinformatic prediction using TargetScan and miRanda identified conserved miR-181a-5p-binding motifs within the 3’ untranslated region (3’-UTR) of OSBPL3. To validate the regulatory relationship between miR-181a-5p and OSBPL3 in MCF-7 cells, qRT-PCR and Western blot analyses were performed following transfection with miR-181a-5p mimics or antisense inhibitors. Overexpression of miR-181a-5p resulted in significant downregulation of both OSBPL3 mRNA and protein levels, whereas miR-181a-5p inhibition conversely upregulated OSBPL3 expression (Figures 3A, 3B). These findings established an inverse correlation between miR-181a-5p and OSBPL3 expression, suggesting miRNA-mediated post-transcriptional regulation. To confirm direct targeting, a dual luciferase reporter assay was conducted using wild-type (WT) and mutant (MUT) OSBPL3 3’-UTR sequences. Co-transfection of miR-181a-5p mimics with the WT 3’-UTR construct into HEK293T cells induced a reduction in luciferase activity, whereas no significant suppression was observed with the MUT 3’-UTR variant (Figure 3C). This sequence-specific interaction conclusively demonstrated that miR-181a-5p directly binds to the 3’-UTR of OSBPL3 to repress its expression in MCF-7 cells.

Has-miR-181a-5p potentially promoted tamoxifen chemosensitivity by negatively regulating OSBPL3 via RAS signaling

**Figure 4:**
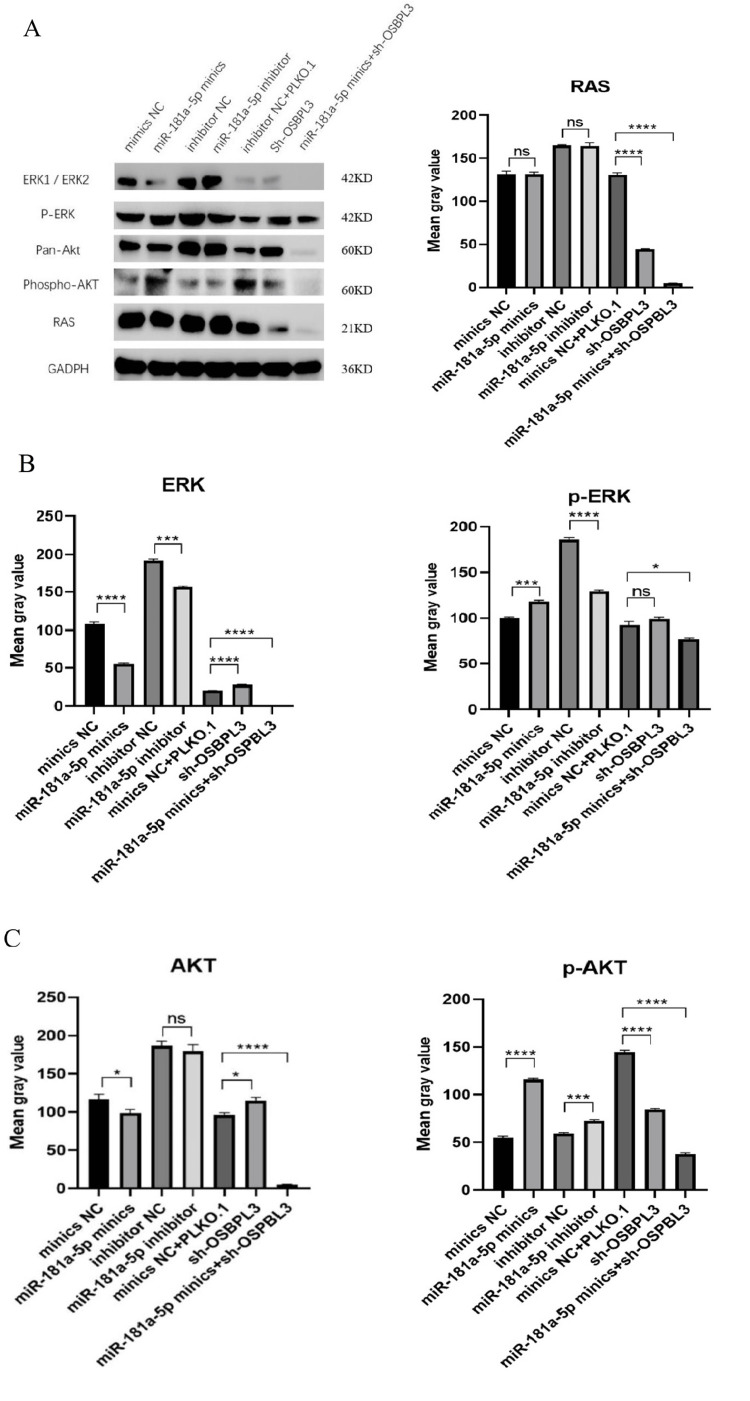
miR-181a-5p affected proliferation, invasion, and apoptosis of breast cancer cells through regulation of RAS signaling pathway. RAS, ERK, phosphorylated ERK (p-ERK), AKT, and phosphorylated AKT (p-AKT) expression in cells was determined by Western blotting. Data reported as mean ± standard deviation of three independent experiments. * P < 0.05, *** P < 0.001, and **** P < 0.0001 vs. control group.

OSBPL3, a multifunctional protein implicated in lipid metabolism, vesicular trafficking, and signal transduction, was identified as a direct downstream target of miR-181a-5p. To uncover the mechanistic basis of OSBPL3-mediated tamoxifen chemosensitization in MCF-7 cells, bioinformatic analysis revealed a significant association with RAS signaling pathways. Results demonstrated that miR-181a-5p may regulate BC cell migration, invasion, and apoptosis through the RAS pathway. Following transfection of MCF-7 cells with miR-181a-5p mimics or antisense inhibitors, Western blotting was employed to quantify RAS signaling effectors. Inhibition of miR-181a-5p markedly upregulated RAS, total ERK, phosphorylated ERK (p-ERK), and total AKT expression compared to mimic-treated cells. Conversely, OSBPL3 knockdown attenuated RAS pathway activation, with significant reductions in RAS, ERK, p-ERK, and phosphorylated AKT (p-AKT) levels versus negative controls (Figure 4). Notably, dual modulation combining miR-181a-5p overexpression and OSBPL3 silencing produced synergistic suppression of RAS signaling. These findings collectively demonstrate that miR-181a-5p potentiates tamoxifen sensitivity in MCF-7 cells through OSBPL3-dependent RAS pathway inactivation. The inverse regulatory axis between miR-181a-5p and OSBPL3 modulates critical oncogenic signaling nodes, including ERK/MAPK and PI3K/AKT cascades, to restore chemotherapeutic responsiveness.

## Discussion

BC remains the second leading cause of cancer-related mortality in women globally [20]. This study elucidates the mechanistic role of miR-181a-5p in modulating tamoxifen resistance in BC cells. A lentiviral-mediated stable transduction system was employed to generate OSBPL3-knockdown BC cells, ensuring sustained interference efficacy [21]. Among three validated shOSBPL3 constructs, shOSBPL3-2 was selected for subsequent studies based on its superior knockdown efficiency and enhanced chemosensitization potential. Mechanistically, MCF7 cells were transfected with miR-181a-5p mimics, scramble mimic controls, siRNA, or non-targeting siRNA, followed by 48-hour tamoxifen exposure. At the pharmacologically active concentration of 5 µM tamoxifen, miR-181a-5p overexpression significantly suppressed cell viability, whereas antisense inhibition partially restored proliferative capacity. Functional phenotyping revealed that miR-181a-5p suppression enhanced metastatic potential, increasing invasion and migration. Conversely, miR-181a-5p mimic transfection augmented tamoxifen-induced apoptosis, while miR-181a-5p inhibition attenuated apoptotic response. Collectively, these findings demonstrate that miR-181a-5p upregulation potentiates tamoxifen efficacy through enhancing drug-induced apoptosis and suppressing proliferative and invasive capacities in BC cells.

Emerging evidence has established OSBPL3 as a critical mediator of oncogenesis across multiple malignancies [10,22,23]. Our study extends this paradigm by identifying a novel inverse regulatory relationship between OSBPL3 and miR-181a-5p in MCF-7 BC cells. Dual luciferase reporter assays conclusively demonstrated that miR-181a-5p was directly bound to the 3’-UTR of OSBPL3 mRNA, mechanistically linking this miRNA to post-transcriptional regulation of its target. To functionally validate OSBPL3’s role in tamoxifen chemoresistance, siRNA-mediated silencing was performed alongside miR-181a-5p modulation. Combined transfections revealed dose-dependent suppression of OSBPL3 expression via qRT-PCR and immunoblotting, with maximal knockdown observed in the combinatorial miR-181a-5p mimic and OSBPL3 siRNA cohort. Functional assays demonstrated that OSBPL3 knockdown significantly suppressed metastatic potential, as evidenced by a reduction in wound closure rate and inhibition of Transwell invasion compared with the controls. Furthermore, OSBPL3 silencing amplified tamoxifen-induced apoptosis, an effect potentiated by miR-181a-5p co-overexpression. These data established OSBPL3 as a key mediator of miR-181a-5p-driven chemosensitization, with synergistic effects arising from coordinated miRNA-mRNA targeting.

Ras, a phylogenetically conserved regulator of proliferation, differentiation, and apoptosis [24], exerts its oncogenic effects through downstream effectors [25],including the PI3K/Akt and Raf/MEK/ERK cascades [26]. PI3K/Akt activation suppresses mitochondrial calcium efflux, a critical apoptosis trigger [27], while ERK phosphorylation drives transcriptional reprogramming to sustain survival [28]. Our studies revealed that OSBPL3 knockdown markedly downregulated Ras signaling-related molecules, with significant reductions in Ras, ERK, phosphorylated ERK, Akt, and phosphorylated Akt. Notably, miR-181a-5p overexpression synergized with OSBPL3 silencing, achieving greater suppression of p-ERK than either intervention alone. These findings position the miR-181a-5p/OSBPL3 axis as a critical modulator of Ras pathway activation, wherein OSBPL3 ablation disrupts oncogenic signaling to restore tamoxifen sensitivity. miR-181a-5p/OSBPL3/Ras signaling axis governs tamoxifen responsiveness in hormone receptor-positive BC. Therapeutic strategies combining miR-181a-5p restoration with OSBPL3 inhibition may offer novel avenues to overcome endocrine resistance. However, our research did not delve deeply into the mechanisms of the RAS pathway, which is a shortcoming on our part.

## Conclusions

In summary, our findings suggested that miR-181a-5p enhanced tamoxifen chemosensitivity in MCF7 BC cells through OSBPL3 suppression via RAS/MAPK and PI3K/AKT signaling axis inhibition. The observed inverse regulatory relationship between miR-181a-5p and OSBPL3 aligns with prior evidence implicating OSBPL3 as an oncogenic driver via RAS pathway activation. miR-181a-5p/OSBPL3 regulatory axis emerges as a promising therapeutic target for overcoming tamoxifen resistance in hormone receptor-positive BC.

## Appendices

**Table 1:** The oligonucleotide sequences.

Name	Sequences
hOSBPL3 qRT F	5'-AATGCCCATGAGATTGAAGG-3'
hOSBPL3 qRT R	5'-CTCGTAGCCTTTCGGCATAG-3'
hOSBPL3 qRT F1	5'-GCCATATCAGCGTATGCATCT-3'
hOSBPL3 qRT R1	5'-AAACTGGAAGCCCTTGTCCT-3'
hOSBPL3 qRT F2	5'-CCACTTTTTCTCAGGGTCCA-3'
hOSBPL3 qRT R2	5'-TGGAACTCGTGTCTCACCAC-3'
miR-181a-5p qRT F	5'-AGCCAACATTCAACGCTGTCG-3'
miR-181a-5p qRT R	5'-CAGTGCAGGGTCCGAGGTATTC-3'
U6 F	5'-AAAGCAAATCATCGGACGACC-3'
U6 R	5'-GTACAACACATTGTTTCCTCGGA-3'
miR-181a-5p RT	5'-GTCGTATCCAGTGCAGGGTCCGAGGTATTCGCACTGGATACGACACTCAC-3'
miR-181a-5p mimic	5'-AACAUUCAACGCUGUCGGUGAGUUCACCGACAGCGUUGAAUGUUUU-3'
miR-181a-5p mimic NC	5'-UUCUCCGAACGUGUCACGUTTACGUGACACGUUCGGAGAATT-3'
miR-181a-5p inhibitor	5'-ACUCACCGACAGCGUUGAAUGUU-3'
miR-181a-5p inhibitor NC	5'-CAGUACUUUUGUGUAGUACAA-3'
OSBPL3 siRNA1	5'-GCCTTTGCCATATCAGCGTAT-3'
OSBPL3 siRNA2	5'-GAAGCGTAGCAGTATATCAAA-3'
OSBPL3 siRNA3	5'-GCTGGAAGCAAGCCATTTAAT-3'
OSBPL3 siRNA NC	5'-TTCTCCGAACGTGTCACGT-3'
